# Serial Circulating Tumor DNA Detection Using a Personalized, Tumor-Informed Assay in Esophageal Adenocarcinoma Patients Following Resection

**DOI:** 10.1053/j.gastro.2021.07.011

**Published:** 2021-11

**Authors:** Emma Ococks, Shruti Sharma, Alvin Wei Tian Ng, Alexey Aleshin, Rebecca C. Fitzgerald, Elizabeth Smyth

**Affiliations:** Medical Research Council Cancer Unit, Hutchison/Medical Research Council Research Centre, University of Cambridge, Cambridge, United Kingdom; Research and Development Department, Natera, Incorporated, San Carlos, California; Cancer Research UK Cambridge Institute, University of Cambridge, Cambridge, United Kingdom; *OCCAMS CONSORTIUM*, Medical Research Council Cancer Unit, Hutchison/Medical Research Council Research Centre, University of Cambridge, Cambridge, United Kingdom; Medical Affairs, Oncology, Natera, Incorporated, San Carlos, California; Medical Research Council Cancer Unit, Hutchison/Medical Research Council Research Centre, University of Cambridge, Cambridge, United Kingdom; 5 Medical Oncology, Cambridge University Hospitals, National Health Service Foundation Trust, Addenbrooke’s Hospital, Cambridge, United Kingdom

Adenocarcinoma of the esophagus is rapidly increasing in incidence.^1^ Esophageal adenocarcinoma (EAC) is frequently advanced at presentation, and even when treated with multimodality therapy, is cured in less than 50% of operated-on patients.^2^^,^^3^

Circulating tumor DNA (ctDNA) has shown promise as a prognostic tool in multiple cancers and is a predictive biomarker for treatment in non-small cell lung cancer.^4^^,^^5^ We recently confirmed the prognostic value of ctDNA using a non-EAC–specific panel in a large population of resected EAC.^6^ In brief, patients who were ctDNA-positive after resection had worse survival than ctDNA-negative patients (hazard ratio, 5.55; 95% confidence interval, 2.42-12.71; *P* = .0003).^6^ However, the sensitivity of a tumor-naïve panel for detecting recurrence was only 35%, implying many patients who recur are not detected.^6^ In this study, we tested whether a personalized, tumor-informed assay would demonstrate superior sensitivity for detecting minimal residual disease (MRD) in patients with resected EAC.

In this retrospective study, blood samples were collected from 20 patients with EAC who underwent surgery or endoscopic mucosal resection (EMR). Blood samples were collected before and after surgical treatment. This study was conducted in accordance with the International Conference on Harmonization-Good Clinical Practice Guidelines and approved by the United Kingdom National Ethics Framework (LREC, 10-H0305-1). All patients provided written informed consent.

We identified tumor-specific variants using whole-genome sequencing data from our International Cancer Genome Consortium project, mean coverage: 73x (tumor) and 37x (blood reference).^7^ We then used 16 of these patient-specific somatic single-nucleotide variants to design individualized multiplex polymerase chain reaction-based primers for next-generation sequencing, used to identify ctDNA in patient plasma.

For survival analysis, only patients who underwent surgery were included. Patients who underwent EMR were expected to be cured and were excluded. Survival estimates were calculated using the Kaplan-Meier method, and survival plots were created using “survminer” R 0.4.4 software (R Foundation for Statistical Computing). Survival differences were evaluated by univariate Cox regression analysis using the “survival” R 2.44-1.1 package. *P* values were determined using the log-likelihood test.

At least 1 sample was taken from all patients before and after tumor removal (Figure 1*A*).

**Figure 1:**
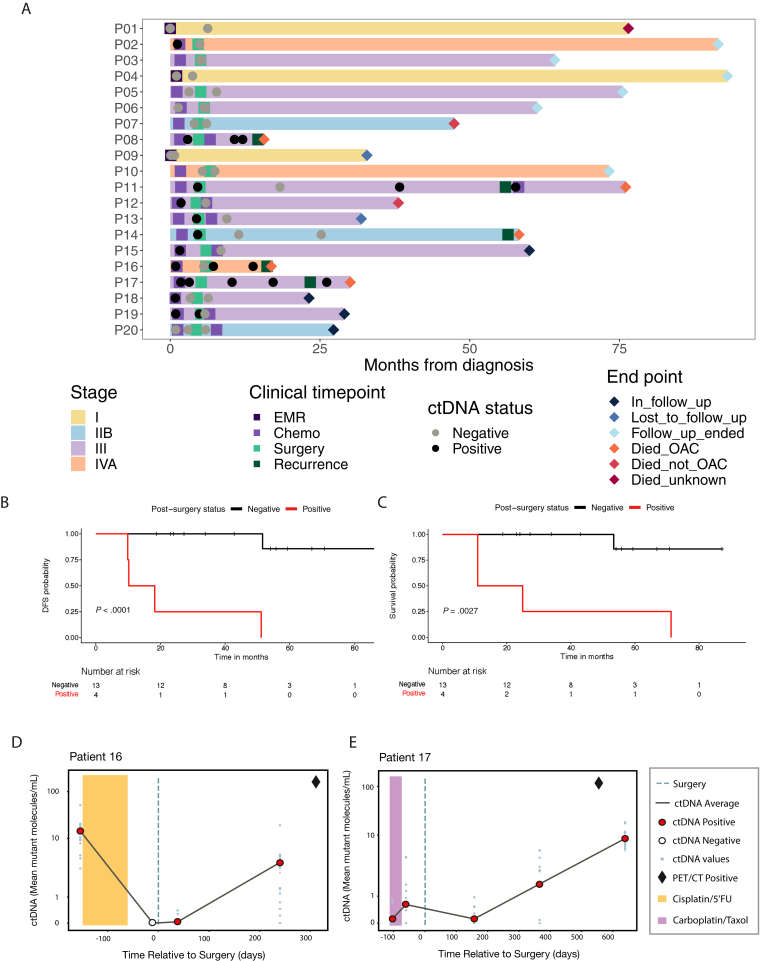
(*A*) Sample timelines of the 20 patients (P) in the cohort. (*B*) Disease-free survival (DFS) in patients according to circulating tumor (ct)DNA status post-surgery. (*C*) Cancer-related survival in patients according to postsurgical ctDNA status. (*D*) Patient who had a good response to chemotherapy, tumor regression grade 1, lead time on patient, 278 days. (*E*) Patient remained ctDNA-positive throughout treatment, and lead time was >500 days. CT, computed tomography; EMR, endoscopic mucosal resection; OAC, oesophageal adenocarcinoma; PET, positron emission tomography; 5′FU, 5′-fluorouracil.

Patient characteristics were consistent with those expected in patients with EAC (median age, 62 years; 85% men) (Supplementary Table 1). Most (17 of 20 [85%]) were treated with perioperative chemotherapy.

Patients with deeper penetration of the gastroesophageal mucosa were more likely to have ctDNA identified preoperatively (9 of 12 [75%] cT3 vs 2 of 5 [40%] T2); however, groups were similar with respect to cN, yN, and lymphovascular invasion (Supplementary Figure 1*A*). All patients that recurred were ctDNA-positive at baseline (100% sensitivity, *P* < .0001) (Supplementary Figure 1*B*). Patients who were ctDNA-positive before surgery had significantly poorer disease-free survival (DFS) (*P* = .042), with a median DFS of 32.0 months vs 63.0 months in ctDNA-negative preoperative patients. There was also a trend towards poorer cancer-specific survival (Supplementary Figure 1*C* and *D*). None of the presurgical ctDNA-negative patients relapsed after surgery (Supplementary Figure 1*C*). Of the 11 presurgical ctDNA-positive patients, 5 (45%) relapsed after surgery.

Four patients were ctDNA-positive after surgery and relapsed, 1 patient, who was ctDNA-negative, developed recurrence 2.6 years after the last ctDNA testing, leading to a sensitivity of 80% (4 of 5) and specificity of 100% (12 of 12). Median DFS was 14.2 months vs 51.2 months in ctDNA-positive vs ctDNA-negative in postoperative patients, respectively (Figure 1*B*), and median cancer-specific survival was 18.0 months vs 53.4 months (Figure 1*C*). ctDNA-positivity at this time point was associated with inferior DFS (*P* < .0001).When patients who did not have a plasma sample within 1 year of relapse were excluded, sensitivity and specificity were 100%. The median ctDNA variant allele fraction detected in positive samples after surgery was 0.01% (range, 0.001%-15.9%). Response to neoadjuvant chemotherapy was reflected in the ctDNA fraction; a patient with a complete response to neoadjuvant chemotherapy was ctDNA-negative after treatment (Figure 1*D*). In contrast residual disease was detected in patients who had a poor response to neoadjuvant chemotherapy, including a patient where the ctDNA fraction increased during treatment (Figure 1*E*).

To our knowledge, this study is the first to investigate the use of a tumor-informed ctDNA assay to detect MRD in resected EAC. We demonstrate excellent sensitivity and specificity of personalized ctDNA assays for the detection of ctDNA in patients after surgical resection. Recurrent disease developed in all patients with ctDNA detected postoperatively. This sensitive ctDNA assay provided a median lead time of almost 1 year before clinical or radiologic recurrence.

One patient who was ctDNA-negative 6 months postoperatively developed a late potentially low ctDNA shedding peritoneal recurrence >4 years after surgery; the last ctDNA sample available for this patient was >2 years before relapse. This implies both temporal and anatomic reasons for the lack of a ctDNA-positive result predicting relapse for this patient. Interestingly, ctDNA preoperatively was modestly prognostic, and this was also associated with tumor stage. Crucially, patients who were ctDNA-positive preoperatively and became ctDNA-negative after surgery had a good prognosis, indicating that ctDNA is a valuable dynamic biomarker.

In colorectal cancer, individualized ctDNA assessment after surgery can be considered a standard of care while the predictive value of such assays is under investigation in large, randomised trials.^8^ In resected EAC, in part due to surgical morbidity, fewer than half of the patients currently undergo the adjuvant component of perioperative chemotherapy.^3^ The benefit of reserving adjuvant chemotherapy for patients most likely to recur or switching to an alternative regimen should be evaluated prospectively. In addition, personalized ctDNA detection could also provide insight on the most suitable treatment option for the patient based on their ctDNA levels after neoadjuvant chemotherapy. Our study also suggests that longitudinal monitoring of ctDNA rather than a sample at a single time point could be valuable, because a minority of patients may have late recurrences.

This study is limited by modest sample size; however, given the robust, individualized methodology of our approach, we believe that these results are likely to be generalizable.

In summary, this study demonstrates that personalized ctDNA assays provide a tool with potential clinical application to predict relapse in patients with resected EAC. The next step will be to design prospective clinical trials that risk stratify adjuvant therapy based on MRD.

## Figures and Tables

**Supplementary Figure 1:**
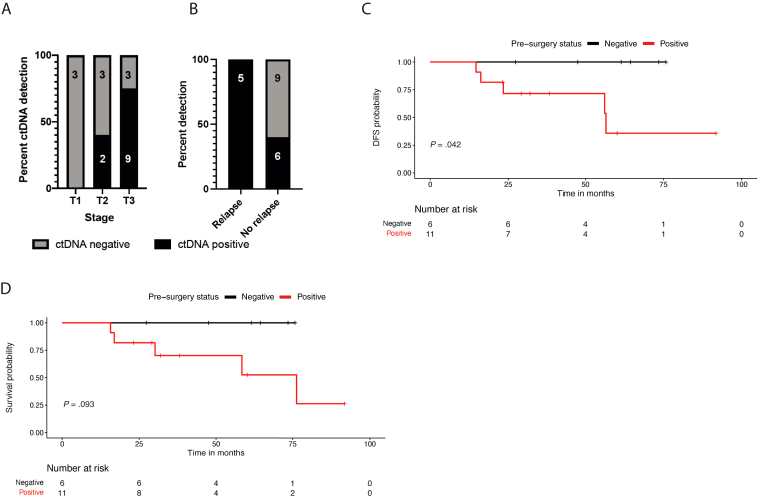
(*A*) Presurgical detection of circulating tumor (ct)DNA across different stages. (*B*) Presurgical detection of ctDNA according to relapse status. (C) Disease-free survival (DFS) in patients according to ctDNA status at baseline. (*D*) Cancer-related survival in patients according to baseline ctDNA status.

**Supplementary Table 1:** Clinical Demographics of Cohort

Variable	No. or Median	% or Range
	(N = 20)	
Sex		
Male	17	85
Female	3	15
Age, *y*	62.8	48.9–80.8
T stage		
T1a	2	10
T1	1	5
T2	5	25
T3	12	60
N stage		
N0	9	45
N1	6	30
N2	2	10
N3	1	5
Nx	2	10
Treatment		
Surgery	17	85
Endoscopic mucosal resection	3	15
Chemotherapy		
Yes	17	85
No	3	15
Siewert’s classification		
1	12	60
2	5	25
3	3	15
